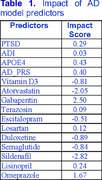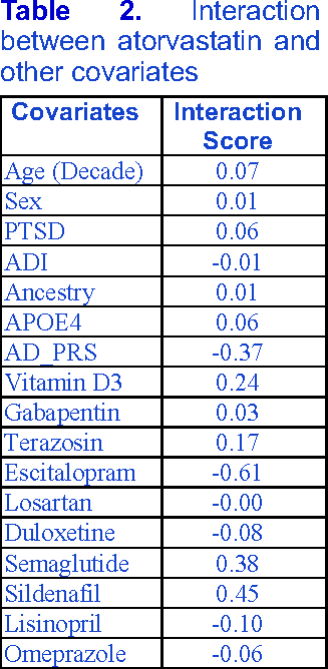# A pilot medication‐wide association study for ADRD drug repurposing using the Million Veteran Program

**DOI:** 10.1002/alz70859_106801

**Published:** 2025-12-26

**Authors:** `Qing Zeng, Edward Zamrini, Ali Ahmed, Debby W Tsuang, Mark W. Logue, Yan Cheng, Yijun Shao, Philip Ma

**Affiliations:** ^1^ George Washington University, Washington, DC USA; ^2^ Washington DC VA Medical Center, Washington, DC USA; ^3^ Irvine Clinical Research, Irvine, CA USA; ^4^ DC VA, Washington, DC USA; ^5^ VA Puget Sound, Seattle, WA USA; ^6^ Department of Psychiatry, Boston University Chobanian & Avedisian School of Medicine, Boston, MA USA; ^7^ VA Washington DC Healthcare, Washington, DC USA

## Abstract

**Background:**

A pilot medication‐wide association study (MWAS) was conducted to identify candidate drugs for repurposing in the prevention of Alzheimer’s disease and related disorders (ADRD). This MWAS is a hypothesis‐free, agnostic exploration of the Million Veteran Project (MVP) dataset, which contains extensive information on genotypes, drug exposure, and incident ADRD.

**Methods:**

Using an age‐, sex‐, and race‐matched cohort (*n* = 263,256) from the MVP, we trained a Histogram‐Based Gradient Boosting (HGB) model incorporating PTSD status, Social Determinants of Health (SDOH) (represented by ADI scores based on patients' most recent zip codes), APOE ε4 status, and Polygenic Risk Scores (PRS) for ADRD. Other predictors included age, gender, ancestry (derived from genomic data), and scaled cumulative doses of vitamin D3, atorvastatin, gabapentin, and other medications. To assess the contribution of individual features to population‐wide ADRD risk, we applied novel explainable AI methods that we developed and validated, calculating an impact score that quantifies each feature’s contribution to ADRD risk, comparable to odds ratio. Additionally, we calculated interaction scores for atorvastatin in relation to other model features, enabling a more nuanced analysis of drug‐drug and drug‐risk factor interactions.

**Results:**

Our analysis confirmed previous findings and aligned with existing literature, suggesting both positive and negative associations between certain medications and ADRD risk. For example, atorvastatin had a negative impact score, indicating a decreased risk of ADRD. Additionally, atorvastatin showed a positive interaction with age, suggesting that its beneficial effect diminishes with increasing age—consistent with prior literature. We also observed a novel negative interaction between atorvastatin and escitalopram, indicating that their concurrent use may reduce ADRD risk beyond the additive effect. This finding is novel and plausible, as both drugs have been reported to lower ADRD risk. The impact and interaction scores for atorvastatin are presented in Tables 1 and 2.

**Conclusion:**

These findings demonstrate the robustness of the MWAS approach while highlighting new insights through the integration of genomic and SDOH data. It also underscores the potential of leveraging real‐world data and explainable AI to identify candidate drugs for ADRD prevention.